# Long non-coding RNA lncC11orf54-1 modulates neuroinflammatory responses by activating NF-κB signaling during meningitic *Escherichia coli* infection

**DOI:** 10.1186/s13041-021-00890-8

**Published:** 2022-01-03

**Authors:** Bojie Xu, Ruicheng Yang, Bo Yang, Liang Li, Jiaqi Chen, Jiyang Fu, Xinyi Qu, Dong Huo, Chen Tan, Huanchun Chen, Zhong Peng, Xiangru Wang

**Affiliations:** 1 State Key Laboratory of Agricultural Microbiology, College of Veterinary Medicine, Huazhong Agricultural University, Wuhan, Hubei China; 2 Key Laboratory of Preventive Veterinary Medicine in Hubei Province, The Cooperative Innovation Center for Sustainable Pig Production, Wuhan, Hubei China; 3 Key Laboratory of Development of Veterinary Diagnostic Products, Ministry of Agriculture of the People’s Republic of China, Wuhan, Hubei China; 4 International Research Center for Animal Disease, Ministry of Science and Technology of the People’s Republic of China, Wuhan, Hubei China

**Keywords:** LncC11orf54-1, Neuroinflammation, Brain microvascular endothelial cells, NF-κB signaling, Meningitic *Escherichia coli*, Blood–brain barrier

## Abstract

*Escherichia coli* is the most common gram-negative pathogenic bacterium causing meningitis. It penetrates the blood–brain barrier (BBB) and activates nuclear factor kappa B (NF-κB) signaling, which are vital events leading to the development of meningitis. Long non-coding RNAs (lncRNAs) have been implicated in regulating neuroinflammatory signaling, and our previous study showed that *E. coli* can induce differential expression of lncRNAs, including lncC11orf54-1, in human brain microvascular endothelial cells (hBMECs). The hBMECs constitute the structural and functional basis for the BBB, however, it is unclear whether lncRNAs are involved in the regulation of inflammatory responses of hBMECs during meningitic *E. coli* infection. In this study, we characterized an abundantly expressed lncRNA, lncC11orf54-1, which was degraded by translocated coilin to produce mgU2-19 and mgU2-30 in hBMECs during *E. coli* infection. Functionally, lncC11orf54-1-originated non-coding RNA mgU2-30 interacted with interleukin-1 receptor-associated kinase 1 (IRAK1) to induce its oligomerization and autophosphorylation, thus promoting the activation of NF-κB signaling and facilitating the production of pro-inflammatory cytokines. In summary, our study uncovers the involvement of lncC11orf54-1 in IRAK1–NF-κB signaling, and it functions as a positive regulator of inflammatory responses in meningitic *E. coli*-induced neuroinflammation, which may be a valuable therapeutic and diagnostic target for bacterial meningitis.

**Supplementary Information:**

The online version contains supplementary material available at 10.1186/s13041-021-00890-8.

## Introduction

Bacterial meningitis is an important life-threatening infection of the central nervous system (CNS), with high morbidity and mortality worldwide. *Escherichia coli* is the most common causative gram-negative pathogenic bacterium [1]. *E. coli* causes CNS dysfunction by penetrating the blood–brain barrier (BBB), inducing local inflammation, increasing BBB permeability, and allowing leukocytes to migrate across the BBB [2, 3]. Brain microvascular endothelial cells (BMECs) constitute the structural and functional basis for the BBB [1, 4]; their invasion by *E. coli* and the activation of inflammatory responses are vital steps in meningitis pathogenesis [5, 6]. Activation of nuclear factor kappa B (NF-κB) signaling, the master regulator of inflammatory responses [7], is a hallmark feature of bacterial meningitis [8]. Although accumulating evidence indicates the involvement of NF-κB signaling in modulating meningitic *E. coli*-induced CNS inflammation [9, 10], our current knowledge on the underlying regulatory mechanisms is limited.

LncRNAs are a novel class of transcripts that are longer than 200 nucleotides, with no or limited protein-coding potential [11]. They regulate diverse biological processes, such as imprinting control, cell differentiation, development, and tumor metastasis via interactions with DNA, RNA, or proteins [12–14]. In the field of immunology, increasing evidences have indicated that lncRNAs have crucial functions in regulating immune responses, including inflammation [15], which involve various inflammation-associated signaling pathways, such as the NF-κB pathway and the mitogen-activated protein kinase (MAPK) pathway [16, 17]. LncRNAs can work in different ways; however, the most studied mechanism is the functioning of lncRNAs as competitive endogenous RNAs to competitively sponge microRNA, thus inhibiting the degradation of mRNA [18]. For example, lncRNA H19 can sponge microRNA let-7a to regulate interleukin-6 (IL-6) expression and increase vascular inflammation [19]. Nevertheless, the regulatory mechanisms of more lncRNAs in meningitic *E. coli*-induced inflammatory responses remain to be systematically and comprehensively elucidated.

In the current study, we characterized an abundantly expressed lncRNA, lncC11orf54-1, in *E. coli-*challenged human BMECs (hBMECs). Meningitic infection in hBMECs by *E. coli* can dramatically degrade lncC11orf54-1 and produce non-coding RNA mgU2-19 and mgU2-30, which positively regulate *E. coli-*triggered pro-inflammatory cytokines production through IL-1 receptor-associated kinase 1 (IRAK1) autophosphorylation-mediated activation of the NF-κB signaling pathway. Thus, lncC11orf54-1 is an important inflammatory regulator of the NF-κB pathway during meningitic *E. coli* infection and may be an important therapeutic and diagnostic target in bacterial meningitis.

## Results

### LncC11orf54-1 displayed differential expression during meningitic *E. coli* infection

**Fig. 1:**
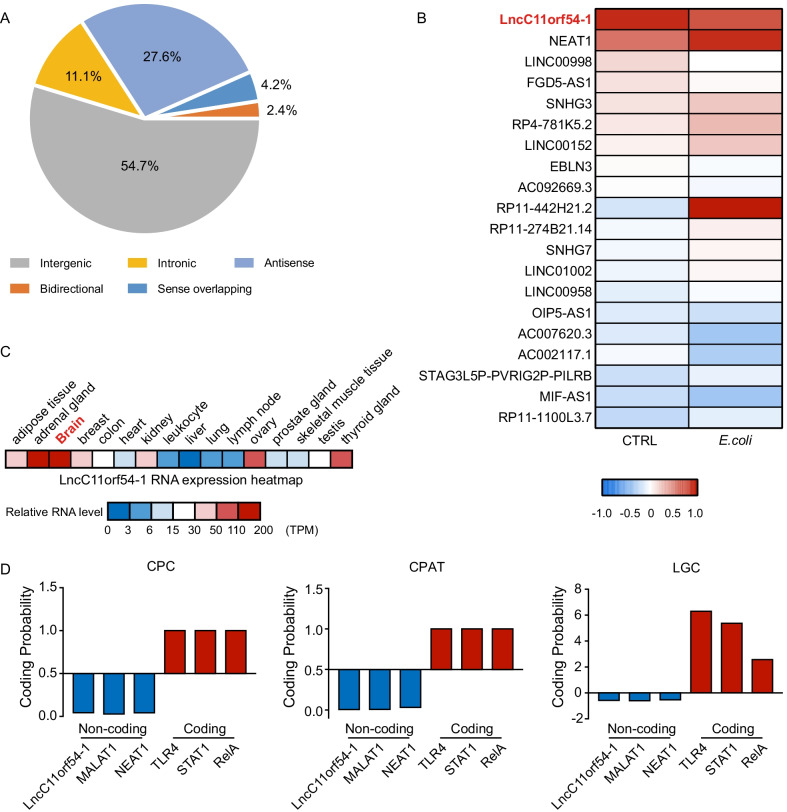
Differentially expressed lncRNAs in hBMECs treated with meningitic *E. coli*. **A** The pie chart shows the different aberrantly expressed lncRNAs in *E. coli* infected hBMECs. **B** Heatmap representing differentially expressed lncRNAs, sorted according to transcript abundance. **C** Heatmap showing relative expression levels of lncC11orf54-1 in different human tissues according to the human Encyclopedia of DNA Elements RNA-seq datasets. **D** Prediction of lncC11orf54-1 coding potential by using CPC, CPAT and LGC

To identify the lncRNAs involved in the inflammatory response induced by meningitic *E. coli*, we performed RNA-seq analysis in hBMECs challenged with or without *E. coli* PCN033 [20]. Reanalysis data showed that 280 known lncRNAs exhibited significant differences in hBMECs in response to meningitic *E. coli* infection (p < 0.05). Among these lncRNAs, 54.7% were mapped to intergenic regions, followed by antisense regions (27.6%), intronic regions (11.1%), sense overlapping regions (4.2%), and bidirectional regions (2.4%) (Fig. 1a). The differentially expressed lncRNAs were subsequently filtered according to their transcript abundance. We identified 20 lncRNAs (Fig. 1b) with this method, among which lncC11orf54-1 was abundantly expressed in hBMECs. LncC11orf54-1 is a 353-nt lncRNA, and tissue distribution analysis in humans revealed that lncC11orf54-1 was abundantly expressed in the brain and adrenal gland (Fig. 1c). Three tools, including the coding potential capacitator (CPC), coding potential, assessment tool (CPAT), ORF length, and GC content (LGC), were subsequently used to predict the protein-coding potential of lncC11orf54-1. The prediction results supported the idea that lncC11orf54-1 has no protein-coding potential, similar to the potential coding analyses of the known lncRNAs MALAT1 and NEAT1, but opposite that of the typical protein-coding mRNAs such as TLR4, STAT1, and RelA (Fig. 1d). Together, these data show that lncC11orf54-1 is a brain-abundant lncRNA that is differentially expressed in hBMECs in response to meningitic *E. coli* infection.

### *E. coli* infection facilitated the degradation of lncC11orf54-1

**Fig. 2:**
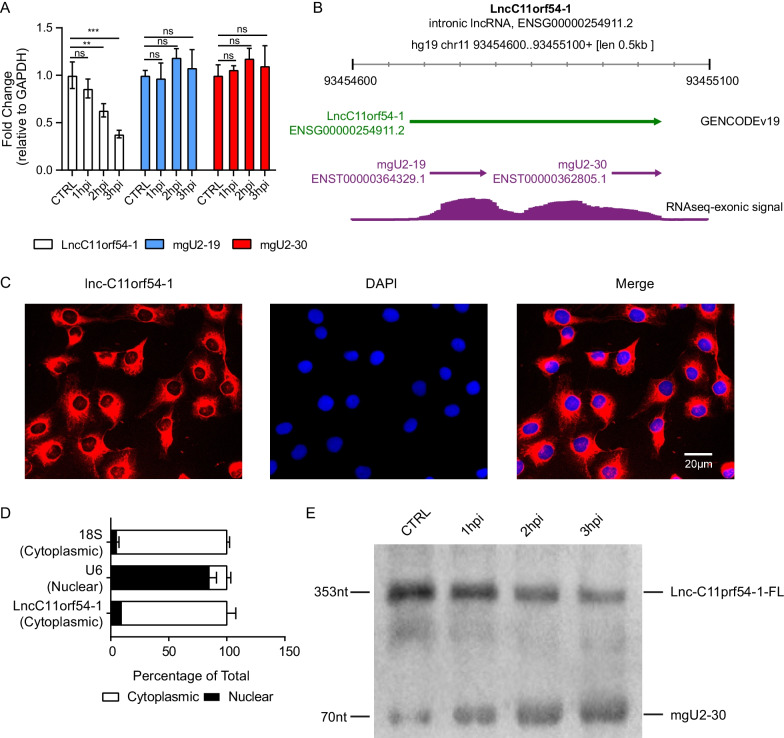
LncC11orf54-1 is downregulated in hBMECs with meningitic *E. coli* infection. **A** Expression of lncC11orf54-1, mgU2-19, and mgU2-30 in hBMECs infected with *E. coli* for 0 h (control, lane 1), 1 h (lane 2), 2 h (lane 3) or 3 h (lane 4) using qPCR analysis. GAPDH was used as the internal reference for the qPCR. Data are presented as mean ± SD from three independent experiments. **p < 0.01, and ***p < 0.001 by student's *t*-test. **B** Visualization of lncC11orf54-1 in the ZENBU browser, showing exonic expression signaling in several different visualizations. **C** Subcellular localization of lncC11orf54-1 (red) in hBMECs by FISH assay. 4′,6-diamidino-2-phenylindole (DAPI) staining is shown in blue. Scale bars, 20 μm. **D** Nuclear/cytoplasmic localization analyses of lncC11orf54-1 in hBMECs by qPCR. The 18S and U6 distribution were selected as the cytoplasmic and nuclear control, respectively. **E** Expression of lncC11orf54-1 and mgU2-30 in hBMECs infected with *E. coli* for 0 h (control, lane 1), 1 h (lane 2), 2 h (lane 3) or 3 h (lane 4) using Northern blotting

The response of lncC11orf54-1 to *E. coli* infection was confirmed by qPCR, and the results showed that lncC11orf54-1 expression was significantly decreased in this process (Fig. 2a). LncC11orf54-1, an intronic lncRNA, is located on chromosome 11 of the human genome. It contains two smaller fragments, known as mgU2-19 and mgU2-30 (Fig. 2b). Determining the subcellular localization of a lncRNA can provide insights into its potential mechanism of action. The subcellular localization of lncC11orf54-1 in hBMECs was determined using nucleocytoplasmic separation and FISH assays. As shown in Fig. 2c, lncC11orf54-1 was primarily located in the cytoplasm. The nucleocytoplasmic separation assay showed that lncC11orf54-1 could be detected in both nuclear and cytoplasmic compartments, but was highly enriched in the cytoplasm, similar to 18S rRNA (Fig. 2d). As shown in Fig. 2a, when challenged with *E. coli*, the expression of lncC11orf54-1 was decreased in hBMECs, while that of mgU2-19 and mgU2-30 was not. To better assess the processed fragments generated on infection of hBMECs with *E. coli*, processing assays were run on formaldehyde agarose gels and Northern blotting. As shown in Fig. 2e, lncC11orf54-1 in hBMECs could be degraded by *E. coli* infection in a time-dependent manner; the expression of full-length lncC11orf54-1 was significantly decreased, while that of the processed mgU2-30 was increased. Taken together, these findings suggest that lncC11orf54-1 is a cytoplasm-located lncRNA in hBMECs and is significantly degraded in response to meningitic *E. coli* infection.

### *E. coli* infection led to the translocation of coilin to the cytoplasm

**Fig. 3:**
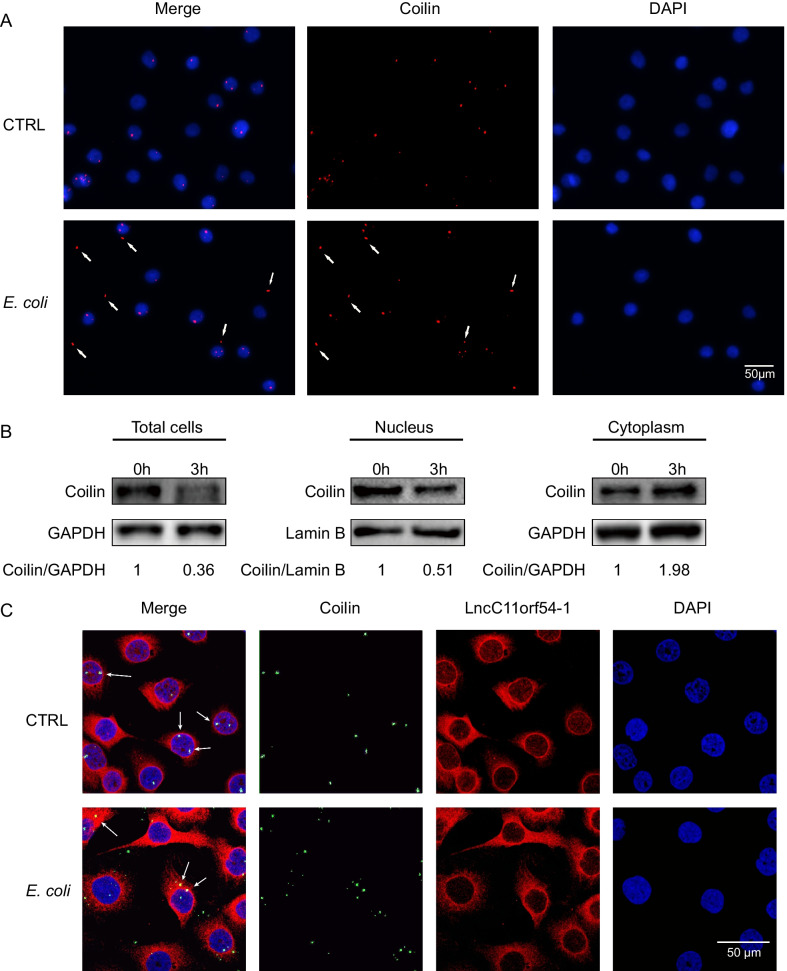
Meningitic *E. coli* infection induces the translocation of coilin. **A** hBMECs were infected with or without meningitic *E. coli*, and the translocation of coilin (red) was detected by fluorescence microscopy. DAPI staining was shown in blue. Scale bars, 50 μm. **B** Nuclear-to-cytoplasmic redistribution analyses of coilin in *E. coli*-stimulated hBMECs. Western blotting analyses of coilin were performed on whole cell lysate, nuclear, and cytoplasmic fractions. GAPDH was used as the loading control for whole-cell lysate and cytoplasmic fractions, whereas for the nucleus fraction, lamin B was used as the loading control. **C** RNA FISH analyses and immunofluorescent analyses determining the co-localization of lncC11orf54-1 (red) and coilin (green) in hBMECs with or without *E. coli* challenge. DAPI staining was shown in blue. Scale bars, 50 μm

Coilin is an RNase generally located in the nucleus, and RNAs like lncC11orf54-1 with GU-rich motif can be the target for coilin processing [21, 22]. Next, an immunofluorescence assay was performed to determine the mechanism underlying the involvement of coilin in the degradation of lncC11orf54-1 in *E. coli-infected* hBMECs. We observed coilin translocation from the nucleus to the cytoplasm upon meningitic *E. coli* infection (Fig. 3a). The nuclear-to-cytoplasmic translocation of coilin was further determined by nucleocytoplasmic separation, followed by Western blotting. We measured the expression of coilin in the nucleus, cytoplasm, and total cells, and the results indicated that *E. coli*-treated hBMECs showed a prominent increase in the levels of cytoplasmic coilin, while levels of total coilin and nuclear coilin were decreased compared with control cells (Fig. 3b), suggesting that *E. coli* induced the transfer of coilin from the nucleus to the cytoplasm or even to the extracellular space. Moreover, RNA FISH and immunofluorescence assay also showed that the translocated coilin colocalized with lncC11orf54-1 in the cytoplasm (Fig. 3c). Thus, these results show that *E. coli* infection promotes the translocation of coilin to the cytoplasm and helps the processing of lncC11orf54-1 in hBMECs.

### The processed product mgU2-30 regulated the activation of NF-κB pathway

**Fig. 4:**
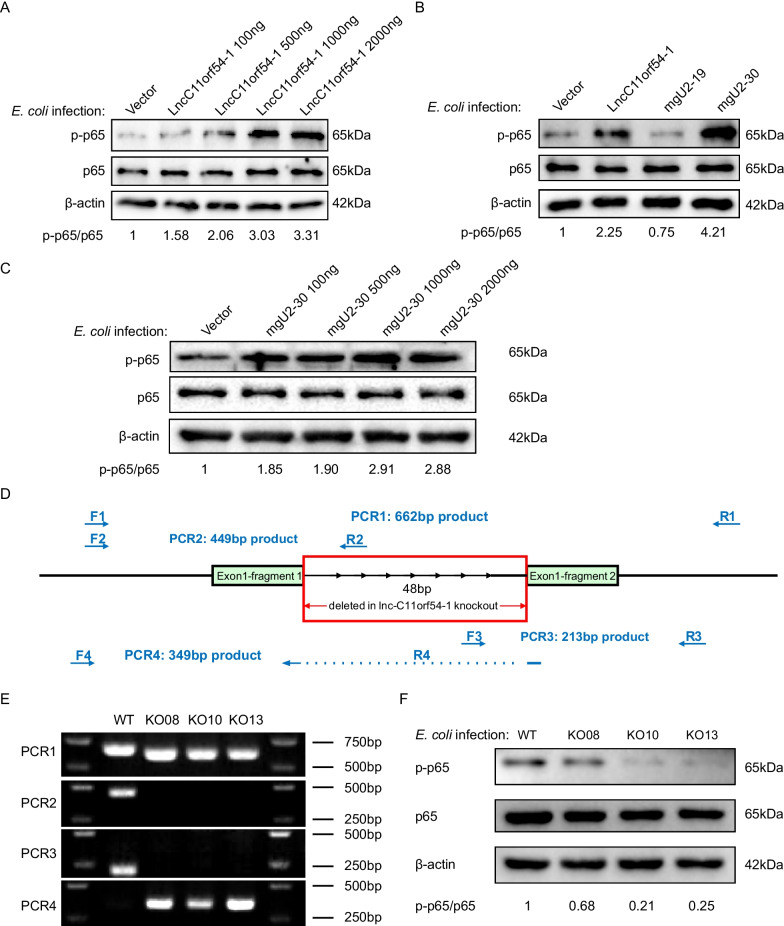
MgU2-30 promotes meningitic *E. coli*-induced activation of NF-κB signaling. **A** Western blot analysis of NF-κB p65 and phosphorylated p65 in hBMECs transfected with multiple dosages of lncC11orf54-1 (0, 100, 500, 1000, and 20,000 ng) under *E. coli* infection. β-actin was used as the loading control. **B** hBMECs were transfected with control construct (pcDNA3.1), overexpression constructs of lncC11orf54-1, mgU2-19, or mgU2-30, and infected with *E. coli*; the protein levels of NF-κB p65 and phosphorylated p65 were determined by Western blotting. β-actin was used as the loading control. **C** Western blot analysis of NF-κB p65 and phosphorylated p65 in hBMECs transfected with multiple dosages of mgU2-30 (0, 100, 500, 1000, and 2000 ng) under *E. coli* infection. β-actin was used as the loading control. **D** Schematic diagram of CRISPR/Cas9 knockout strategies at the mgU2-30 loci. A deletion of 48 bp was validated by sequencing. The red rectangle outlines the deletion region, the blue arrows indicate genotyping PCR primers for identifying knocked-out cell clones. **E** Gel image shows PCR identification results for wildtype hBMECs, and mgU2-30 knockout hBMECs. **F** Effects of mgU2-30 knock out on the expression of NF-κB p65 and phosphorylated p65 in *E. coli* treated hBMECs, as determined by Western blotting. β-actin was used as the loading control

To define the functional role of lncC11orf54-1 in meningitic *E. coli*-induced inflammatory responses in hBMECs, lncC11orf54-1 was overexpressed or knocked out in hBMECs. In *E. coli-*challenged hBMECs, overexpression of lncC11orf54-1 promoted the phosphorylation of the NF-κB p65 subunit in a dose-dependent manner (Fig. 4a). As previously demonstrated, lncC11orf54-1 can be processed to mgU2-19 and mgU2-30 in *E. coli-*infected hBMECs, therefore, we validated the potential regulatory effects of mgU2-19 or mgU2-30 on p65 phosphorylation. In agreement with the lncC11orf54-1 overexpression results, the overexpression of mgU2-30 in hBMECs also dose-dependently enhanced the phosphorylation level of p65, but mgU2-19 had no such effect (Fig. 4b and c).

To further confirm that mgU2-30 functions to activate the NF-κB pathway, we used CRISPR-Cas9 mediated genome editing in hBMECs to generate double-strand DNA breaks in the mgU2-30 region of lncC11orf54-1. Through screening for clones, we retained three clones in which the mgU2-30 region was deleted, and the PCR identification results for these deleted clones are shown in Fig. 4d and e. The results showed that in *E. coli-*infected hBMECs, knockout of the mgU2-30 region of lncC11orf54-1 reduced the phosphorylation level of p65 (Fig. 4f). Collectively, these data indicate that the mgU2-30 fragment generated from lncC11orf54-1 facilitates the activation of the NF-κB pathway in the *E. coli*-induced inflammatory response of hBMECs.

### MgU2-30 promoted IRAK1 oligomerization and facilitated its auto-phosphorylation

**Fig. 5:**
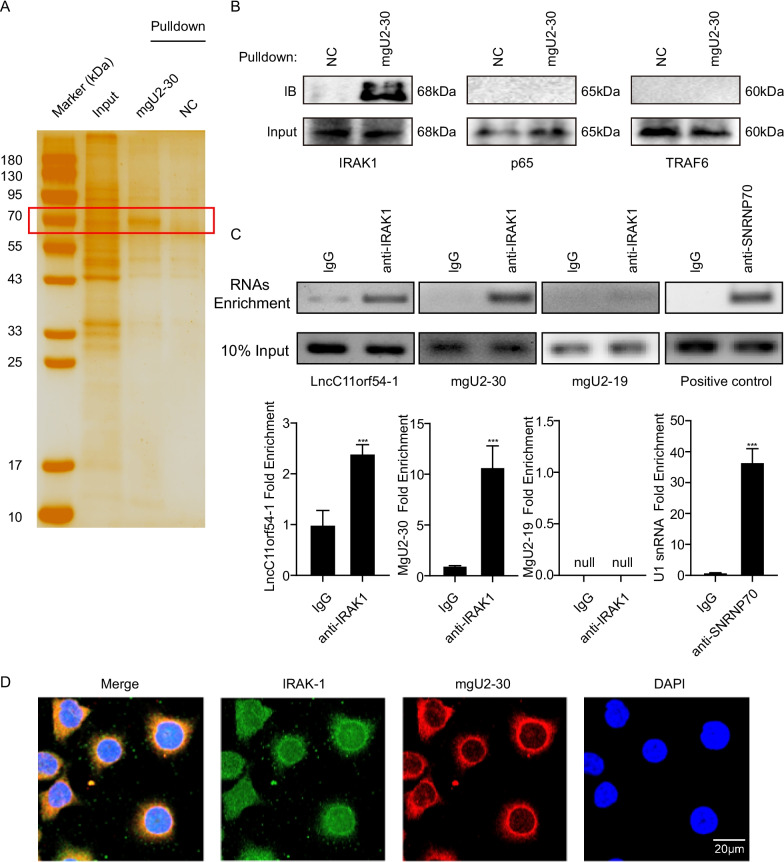
Identification of IRAK1 as a binding protein of mgU2-30. **A** Silver staining of biotinylated mgU2-30-associated proteins in hBMECs. The mgU2-30-specific bands were highlighted with rectangle outlines. **B** Immunoblots of proteins from RAP assay by biotinylated mgU2-30 or negative control probe. **C** IRAK1 RIP followed by PCR assay of purified RNAs from hBMECs. The positive control U1 snRNA PCR product was also observed in the anti-SNRNP70 RIP. Densitometry was performed to analyze differences. “Null” columns represented the absence of densitometry data. Data were presented as mean ± SD from three independent assays. * *p* < 0.05, *** *p* < 0.001. **D** RNA FISH detecting mgU2-30 (red) combined with immunofluorescence staining of IRAK1 (green) in hBMECs. DAPI staining was shown in blue. Scale bars, 20 μm

Although we have provided clues indicating that p65 is a key regulator of the mgU2-30-dependent inflammatory response, the details of the molecular mechanism by which mgU2-30 regulates p65 phosphorylation are unclear. Here, we performed RAP assay with biotinylated mgU2-30 probes, followed by immunoblotting, to characterize the mgU2-30-interacting proteins in hBMECs. The associated proteins were analyzed by SDS-PAGE with silver staining. We found that proteins around 60 kDa may interact with mgU2-30 (Fig. 5a). Three key signaling proteins (IRAK1, p65, and TRAF6) with crucial functions in the activation of the canonical NF-κB pathway were identified by immunoblotting, and among them, IRAK1 was confirmed to bind to mgU2-30 (Fig. 5b). To further verify this interaction between IRAK1 and mgU2-30, we used RIP assay and found that the anti-IRAK1 antibody significantly enriched mgU2-30 (Fig. 5c). Moreover, RNA FISH combined with immunofluorescence demonstrated the colocalization of mgU2-30 and IRAK1 (Fig. 5d and Additional file 1: Fig. S1).

**Fig. 6:**
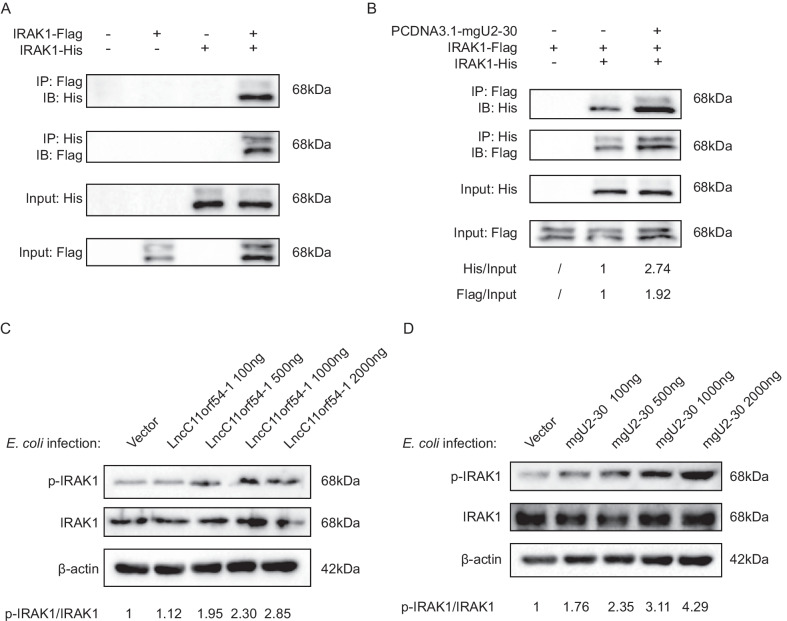
mgU2-30 promotes IRAK1 oligomerization and auto-phosphorylation. **A** Immunoprecipitation of His-IRAK1 (or Flag-IRAK1) followed by immunoblotting (IB) analysis of Flag-IRAK1 (or His-IRAK1) in Flag‐tagged IRAK1 and His‐tagged IRAK1 pair co‐transfected hBMECs (Flag-tagged IRAK1/His-tagged empty vector, His-tagged IRAK1/Flag-tagged empty vector, or Flag-tagged empty vector/His-tagged empty vector pairs were also co-transfected as negative controls). **B** IB analysis of Flag-IRAK1 (or His-IRAK1) of hBMECs transfected with both tagged-IRAK1 constructs after His-IRAK1 (or Flag-IRAK1) immunoprecipitation to detect IRAK1 oligomerization in the presence or absence of mgU2-30. **C** and **D** Western blot analysis of IRAK1 and phosphorylated IRAK1 in hBMECs transfected with multiple dosages of lncC11orf54-1 (**C**) or mgU2-30 (**D**) (0, 100, 500, 1000, and 20,000 ng) under *E. coli* infection. β-actin was used as the loading control

IRAK1 is a serine/threonine kinase that mediates the activation of NF-κB and MAPK pathways [23]. Its oligomerization and auto-phosphorylation are essential for signal transduction, resulting in IRAK1 activation and execution of the pathways [24, 25]. We subsequently determined whether the interaction of mgU2-30 with IRAK1 affected its oligomerization and phosphorylation, and cells were transfected to express Flag-tagged IRAK1 and his-tagged IRAK1, with or without the presence of mgU2-30 overexpression constructs. Co-immunoprecipitation assays demonstrated that IRAK1 was able to interact with itself (Fig. 6a), and this oligomerization of IRAK1 could be promoted by mgU2-30 (Fig. 6b). To determine the effects of the lncC11orf54-1-derived mgU2-30 fragment on the phosphorylation of IRAK1, hBMECs were transfected with a gradient of lncC11orf54-1 overexpression constructs, as shown in Fig. 6c and d. These experiments revealed that overexpression of lncC11orf54-1 in *E. coli*-challenged hBMECs promoted the phosphorylation of IRAK1 in a dose-dependent manner, and mgU2-30 also dose-dependently facilitated IRAK1 phosphorylation. Altogether, these data suggest that mgU2-30 generated from lncC11orf54-1 interacts with IRAK1 to promote its oligomerization, which eventually leads to the auto-phosphorylation of IRAK1.

### MgU2-30 promoted *E. coli*-induced inflammatory responses in hBMECs

**Fig. 7:**
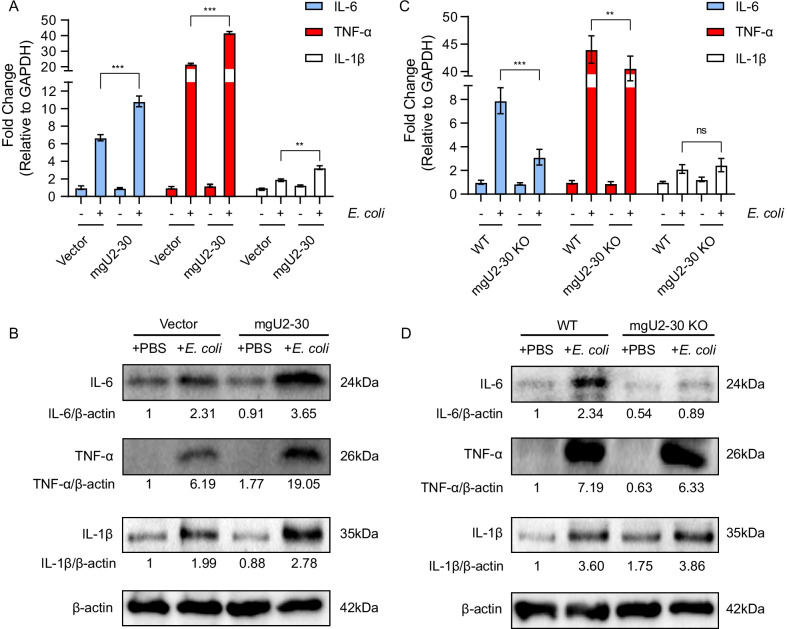
mgU2-30 augments the meningitic *E. coli*-induced inflammatory response in hBMECs. **A** Effects of mgU2-30 overexpression on the expression of inflammatory factors in hBMECs, determined by qPCR. GAPDH was used as the internal reference. Mean ± SD were shown, n = 3. **p < 0.01, and ***p < 0.001 by the student's t-test. **B** Effects of mgU2-30 overexpression on the expression of inflammatory factors in hBMECs, as determined by Western blotting. β-actin was used as the loading control. **C** Effects of mgU2-30 knockout on the expression of inflammatory factors in hBMECs, determined by qPCR. GAPDH was used as the internal reference. Mean ± SD were shown, n = 3. **p < 0.01, and ***p < 0.001 by the t-test. **D** Effects of mgU2-30 knockout on the expression of inflammatory factors in hBMECs, as determined by Western blotting. β-actin was used as the loading control

We further determined the effects of mgU2-30 in hBMECs by overexpressing or knocking out mgU2-30, which were subsequently infected with or without *E. coli*. At 3 h after *E. coli* infection, the qPCR results showed that overexpression of mgU2-30 led to a significant increase in the levels of pro-inflammatory cytokines IL-6, TNF-α, and IL-1β, whereas knockout of mgU2-30 did not cause an increase in the level of these cytokines in hBMECs (Fig. 7a and c). Consistent with the qPCR results, Western blot analysis indicated that mgU2-30 overexpression promoted the expression of pro-inflammatory cytokines in hBMECs under *E. coli* challenge (Fig. 7b). In contrast, the knockout of mgU2-30 in hBMECs no longer caused such a significant increase of these cytokines (Fig. 7d). Moreover, simple overexpression or knockout of mgU2-30 without *E. coli* infection did not result in a change in pro-inflammatory cytokines at either the transcriptional and protein levels (Fig. 7a–d). Taken together, these findings suggest that mgU2-30 can effectively facilitate the expression of pro-inflammatory cytokines in meningitic *E. coli*-infected hBMECs and eventually aggravate the inflammatory response of hBMECs.

## Discussion

**Fig. 8:**
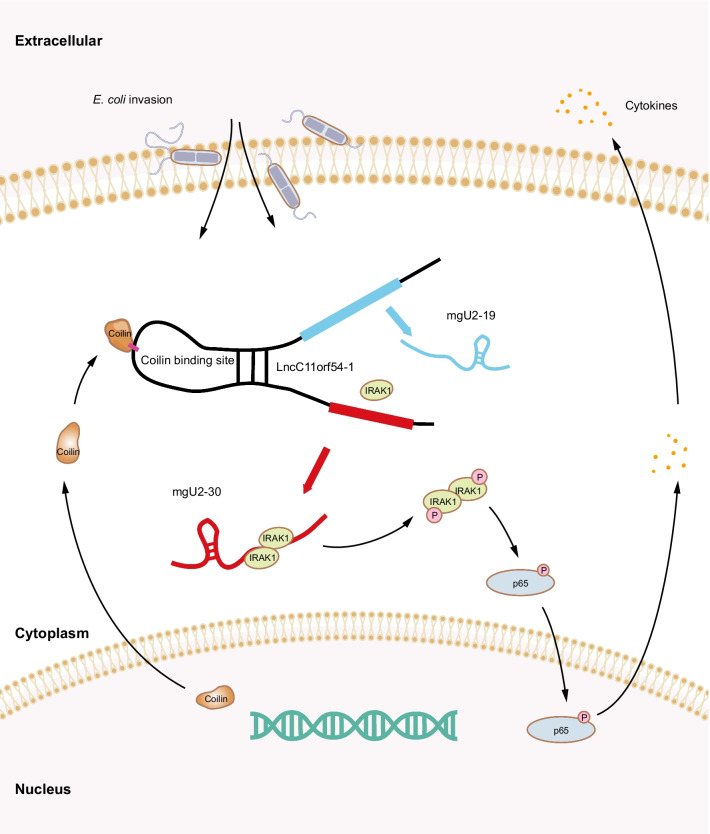
Schematic of the molecular mechanism of lncC11orf54-1 in meningitic *E. coli*-triggered inflammatory responses. Meningitic *E. coli* infection of hBMECs induces the degradation of lncC11orf54-1 and the production of mgU2-30, while mgU2-30 facilitates the oligomerization and phosphorylation of IRAK1, subsequently promoting the inflammatory responses

Accumulating evidence has suggested that lncRNAs are involved in inflammatory processes [16, 26, 27] and in regulating CNS-related diseases, such as ischemic stroke, multiple sclerosis, and Huntington’s disease [28–30]. However, the roles of lncRNAs in CNS infectious diseases remain poorly understood. In this study, we reanalyzed lncRNA expression profiles in response to meningitic *E. coli* challenge in hBMECs and characterized a differentially expressed lncRNA, lncC11orf54-1. *E. coli* infection led to the processing of lncC11orf54-1 into mgU2-30, which effectively augmented the infection-caused inflammatory responses by facilitating the oligomerization and autophosphorylation of IRAK1 (Fig. 8).

In the past decade, lncRNAs have been increasingly recognized as regulators of diverse pathological processes, such as cancer, chronic inflammation, and infectious diseases [31, 32]. Studies have indicated that lncRNAs play regulatory roles in the epigenetic, transcriptional, post-transcriptional, and translational levels and in post-translational modifications [33–35]. Among these, the most reported regulatory mechanism is the action of lncRNAs as competitive endogenous RNAs to competitively sponge miRNAs, thus resulting in decreased mRNA degradation [36]. Over 3000 studies have reported that lncRNAs function as sponges to interact with miRNAs; for example, lncRNA ZFAS1 can sponge miR-150-5p to upregulate VEGFA expression to contribute to the progression of colorectal cancer [37], yet other regulatory mechanisms of lncRNAs are few reported. Our study aimed to uncover the functional lncRNAs involved in human vascular inflammation via a unique mechanism. In *E. coli-*challenged hBMECs, we filtered out lncRNAs with high abundance, which might suggest certain potential biological function. In addition, before finally focusing on lncC11orf54-1, a major of these lncRNAs were overexpressed in hBMECs and this lncRNAs-overexpression screen revealed that lncC11orf54-1 contributed to NF-κB p65 subunit phosphorylation during E. coli infection. We therefore considered lncC11orf54-1 as an effective regulator of inflammatory responses in meningitic *E. coli*-induced neuroinflammation.

LncC11orf54-1 is an intronic lncRNA, also known as SCARNA9, which contains two smaller fragments, mgU2-19 and mgU2-30 and a GU-rich motif between them. Various studies have demonstrated that in vitro transcribed lncC11orf54-1 can be processed to generate mgU2-19 and mgU2-30 by co-incubating with the purified protein coilin [22, 38, 39]. Coilin is a Cajal body marker protein with RNase activity and is generally located in the nucleus. Coilin is occasionally motile; it can make large movements and traverse the full diameter of the nucleus in some cases, such as in cell division [40, 41]. Since previous studies have already shown that coilin can target RNAs with GU-rich motif and degrade them, we further demonstrated that in our hBMECs model, *E. coli* led to the translocation of coilin from the nucleus to the cytoplasm, thus providing the opportunity for coilin to degrade cytoplasmic lncC11orf54-1. And actually, it was observed that lncC11orf54-1 was indeed degraded.

The NF-κB pathway is considered a central mediator of the immune response, and most bacteria can activate the NF-κB pathway. For example, *E. coli* K1 IbeA-binding proteins Vimentin and PTB-associated splicing factor act in concert to activate NF-κB [9], while *Streptococcus pneumoniae* can activate NF-κB by triggering the host factors toll-like receptors 2 and 4 [42]. However, the function of host lncRNAs in activating the NF-κB pathway during bacterial infection is poorly understood. We, thus, indicated that lncRNA lncC11orf54-1 is involved in the phosphorylation of p65, which indicates activation of the NF-κB pathway. There are at least two pathways of NF-κB activation. The canonical pathway relies on IKKγ–IKKβ-mediated degradation of the NF-κB inhibitor alpha (IκBα). The alternative pathway relies on IKKα-mediated p100 phosphorylation and processing to p52 [43, 44]. Among them, IRAK1 is a key signal transducer [25], which can be recruited by myeloid differentiation marker 88 (MyD88) and leads to the phosphorylation of IRAK1 and IRAK4 [45]. Phosphorylation of IRAK1 and IRAK4 facilitates oligomerization and auto-ubiquitination of TRAF6 [46], which is followed by the activation of IKK, and ultimately leads to the activation of NF-κB [47]. A few studies have reported that lncRNAs can influence the modification of these signaling molecules and affect the activation of the NF-κB pathway. For example, lncRNA Mirt2 inhibits the activation of NF-κB and MAPK pathways by attenuating the K63 ubiquitination of TRAF6 in macrophages [16]. In contrast, our results revealed that the product of lncC11orf54 processed by coilin could enrich IRAK1. Since it is generally believed that IRAK1 undergoes oligomerization and autophosphorylation, we subsequently demonstrated that the oligomerization and autophosphorylation of IRAK1 could be promoted by enrichment of mgU2-30, thereby facilitating the activation of the NF-κB pathway.

Inflammation is a fastidiously balanced condition orchestrated by cytokines, chemokines, and their respective receptors [48]. In bacterial meningitis, bacterial invasion across the BBB stimulates BMECs, pericytes, astrocytes, and microglia to release a variety of inflammatory factors, including cytokines and chemokines, resulting in severe CNS inflammatory responses [8, 49, 50]. In the current study, we found that mgU2-30 potentiated the release of the pro-inflammatory cytokines IL-6, TNF-α, and IL-1β, which represented a potential relationship between mgU2-30 and the neuroinflammatory response. Taken together, our findings provide substantial evidence on the pro-inflammatory function of lncRNAs during meningitis-causing bacterial infection. In meningitic *E. coli-*treated hBMECs, we demonstrated that mgU2-30 derived from lncC11orf54-1 could function as a reservoir to enrich IRAK1, which facilitated the oligomerization and auto-phosphorylation of IRAK1 and, therefore, the phosphorylation of p65. Activation of p65 eventually potentiates vascular inflammation. However, inflammation is a complicated and dynamic process, and our in vitro hBMECs model could only partially resemble in vivo conditions, and the effects of different factors on meningitic *E. coli*-induced inflammatory responses should be considered. In addition, further studies are required to explore the mechanisms of coilin translocation caused by *E. coli* infection.

In conclusion, we characterized the function of lncRNA lncC11orf54-1 in meningitic *E. coli*-infected hBMECs and investigated its possible working mechanism. Our data confirmed that *E. coli* infection in hBMECs induced the translocation of coilin, which led to the processing of lncC11orf54-1 into mgU2-30. MgU2-30 in hBMECs specifically facilitated the phosphorylation of IRAK1, thus potentiating inflammatory responses by activating NF-κB (Fig. 8). These findings suggest that lncC11orf54-1 and mgU2-30 may serve as novel targets for future therapeutic strategies against bacterial meningitis. Moreover, the translocation of coilin from the nucleus to the cytoplasm may be a potential target for the diagnosis of inflammation.

## Materials and methods

### Cell line and cell culture

The hBMECs cell line was kindly provided by Prof. Kwang Sik Kim from Johns Hopkins University School of Medicine. It was routinely cultured in RPMI1640 media with 10% fetal bovine serum, 2 mM L-glutamine, 1 mM sodium pyruvate, essential amino acids, non-essential amino acids, vitamins, and penicillin and streptomycin (100 U/mL) in a 37 °C incubator under 5% CO_2_ until the cell monolayer reached confluence.

### Meningitic* E. coli* infection of hBMECs

The meningitic *E. coli* strain PCN033, a highly virulent cerebrospinal fluid isolate isolated in China in 2006, was used as the model [51]. Bacterial cells were routinely grown in Luria–Bertani medium at 37 °C overnight. hBMECs were challenged with *E. coli* PCN033 as follows. An overnight *E. coli* culture was resuspended and diluted in serum-free medium and then added to the starved confluent hBMECs monolayer at a multiplicity of infection (MOI) of 10 (approximately 10^7^ colony-forming units per mL) and incubated at 37 °C with 5% CO_2_ for 3 h.

### Transfection

hBMECs were cultured in six-well plates and grown to 50–60% confluence. Cells were subsequently transfected with the constructs using the jetPRIME transfection reagent (Polyplus transfection, Illkirch, France), according to the manufacturer’s instructions. Briefly, 200 μL of jetPRIME buffer was added to the 2000 ng of plasmids before adding 4 μL of jetPRIME. The suspension was briefly mixed by vortexing, incubated at 25 °C for 10 min, and then added dropwise to the cells and cultured at 37 °C with 5% CO_2_ for 48 h.

### RNA isolation and quantitative real-time polymerase chain reaction analysis

hBMECs were washed three times with ice-cold phosphate-buffered saline (PBS) before RNA extraction. Total RNA was extracted from hBMECs using TRIzol reagent (Invitrogen, Carlsbad, CA, USA) according to the manufacturer’s protocol. After RNA extraction, 500 ng of total RNA was reverse transcribed into cDNA using HiScript II Q RT SuperMix (Vazyme, Nanjing, China). Quantitative real-time polymerase chain reaction (qPCR) was performed using a qTOWER3/G qPCR thermal cycler (Analytikjena, Jena, Germany) with the AceQ qPCR SYBR Green Master Mix (Vazyme), according to the manufacturer’s instructions. The amplification conditions were as follows: 50 °C for 2 min, 95 °C for 10 min, followed by 40 cycles of 95 °C for 15 s and 60 °C for 1 min. The products were then subjected to a melting curve stage comprising denaturation at 95 °C for 15 s, annealing at 60 °C for 1 min, and slow dissociation by ramping from 60 °C to 95 °C at 0.1 °C/s to ensure the specificity of the primers for their target sequences. The relative gene expression was normalized to that of glyceraldehyde 3-phosphate dehydrogenase (GAPDH). The primers used for qPCR are listed in Additional file 1: Table S1. Each assay was performed in triplicates.

### RNA fluorescence in situ hybridization and protein immunofluorescence

For RNA fluorescence in situ hybridization (FISH), a commercial FISH kit was purchased from Bersinbio (Guangzhou, China) and used according to the manufacturer’s instructions. Briefly, hBMECs grown on 20-mm cell culture dishes (SORFA, Huzhou, China) were fixed in 4% paraformaldehyde for 30 min and washed twice with DNase/RNase-free PBS. Cells were permeabilized with 0.1% Triton X-100 for 15 min, washed twice with PBS, and then treated with 2 × saline sodium citrate (SSC). These samples were subsequently treated as follows: 70% ethanol for 5 min, 85% ethanol for 5 min, and 100% ethanol for 5 min. Dried samples were then incubated with pre-hybridization buffer (2 × SSC, 10% formamide) at 37 °C for 30 min, followed by incubation in the hybridization buffer with probes for lncC11orf54-1 (5′-Cy3-CCACCCTCAATCTCATTCAT-3′) and mgU2-30 (5′-Cy3-AGCTCAGGTCAAGTGTAGAA-3′) to a final concentration of 100 nM at 37 °C overnight. The samples were then washed with SSC buffer. All probes were purchased from GenePharma (Shanghai, China). For colocalization analysis of RNA and protein, the RNA FISH protocol was combined with protein immunofluorescence. Samples were treated with blocking buffer and then incubated for 2 h with primary antibodies against IRAK1 (Sigma-Aldrich, St. Louis, MO, USA) or coilin (Proteintech, Chicago, IL, USA), followed by fluorescein isothiocyanate-conjugated goat anti-mouse or anti-rabbit secondary antibody (Biodragon, Beijing, China). Nuclei were counterstained with 4′,6-diamidino-2-phenylindole (Beijing Solarbio Science & Technology Co., Ltd., Beijing, China). Images were acquired using a confocal microscope (LSM710, Carl Zeiss, Oberkochen, Germany).

### Northern blotting

The hBMECs were infected with meningitic *E. coli*, followed by RNA isolation. Fifty micrograms of RNA from each sample were run on 1.5% formaldehyde agarose gel in 5 × 3-morpholinopropane-1-sulfonic acid buffer at 50 V for 60 min. The gel was then washed in diethyl pyrocarbonate-treated H_2_O and 10 × SSC and transferred onto a positively charged nylon membrane by siphoning. After transfer, the membrane was rinsed quickly in distilled water and allowed to dry. The RNA was then cross-linked to the membrane using an ultraviolet cross-linker (UVP, Upland, CA, USA) at a setting of 120,000 μJ/cm^2^. The membrane was placed in a hybridization bottle and pre-hybridized using Ultrahyb Ultrasensitive Hybridization buffer (Ambion Life Technologies, Grand Island, NY, USA) at 42 °C for 1.5 h. The digoxigenin (DIG)-labeled probe for mgU2-30 (5′-DIG-AGCTCAGGTCAAGTGTAGAA-3′) was purchased from Genscript (Nanjing, China). After pre-hybridization, the membrane was hybridized with Ultrahyb Ultrasensitive Hybridization buffer containing a probe at 42 °C overnight in a slow rotating hybridization oven. The blots were then washed with 2 × SSC/0.1% sodium dodecyl sulfate (SDS) and 0.5 × SSC/0.1% SDS and detected using the DIG Nucleic Acid Detection Kit (Roche, Basel, Switzerland) according to the manufacturer’s instructions.

### Western blotting

hBMECs were lysed using radioimmunoprecipitation assay buffer (Epizyme, Shanghai, China) with protease inhibitor cocktail and phosphatase inhibitor cocktail (MedChemExpress, Monmouth, NJ, USA), followed by centrifugation at 12,000 rpm for 15 min at 4 °C to remove insoluble cell debris. The supernatants were measured using the bicinchoninic acid protein assay kit (NCM Biotech, Suzhou, China) and used for western blot analyses. Equal amounts of protein were separated by 12% SDS–polyacrylamide gel electrophoresis (PAGE) and transferred to polyvinylidene difluoride membranes. Membranes were blocked with 5% bovine serum albumin in Tris-buffered saline with Tween 20, followed by immunoblotting with primary antibodies against coilin, GAPDH, lamin B, β-actin, IL-6, tumor necrosis factor-α (TNF-α), IL-1β, His, Flag (Proteintech, Chicago, IL, USA), IRAK1, TNF receptor-associated factor 6 (TRAF6) (Merck Millipore, Billerica, MA, USA), NF-κB p65, phospho-NF-κB p65 (Cell Signaling Technology, Danvers, MA, USA), or phospho-IRAK1 (Abcam, Cambridge, MA, USA). Membranes were then washed and incubated with horseradish peroxidase-conjugated anti-rabbit or anti-mouse secondary antibodies (Biodragon, Beijing, China). The blots were visualized with the Super electrochemiluminescence Prime kit (US Everbright, Suzhou, China) and densitometrically analyzed using Image Lab software (Bio-Rad, Hercules, CA, USA).

### RNA immunoprecipitation

The RNA immunoprecipitation (RIP) assay was performed using the Magna RIP RNA-Binding Protein Immunoprecipitation Kit (Merck Millipore, Billerica, MA, USA), according to the manufacturer’s instructions. Briefly, protein A/G magnetic beads were incubated with anti-IRAK1 antibody, SNRNP70 antibody (positive control), or mouse IgG (negative control), and rotated for 30 min at room temperature. Antibody-treated magnetic beads were then co-incubated with hBMECs lysates and rotated overnight at 4 °C, followed by digestion with proteinase K buffer and purification of RNA. The purified RNA was further quantified and reverse transcribed into cDNA. The abundance of target genes was determined by qPCR.

### RNA antisense purification

The RNA antisense purification (RAP) assay was performed using the RNA Antisense Purification Kit (BersinBio, Guangzhou, China) according to the manufacturer’s instructions. Briefly, hBMECs were cross-linked and homogenized in the presence of protease and RNase inhibitors, and DNA was subsequently removed using DNase. Samples were denatured and incubated with biotin-labeled probes for mgU2-30 (5′-Biotin-AGCTCAGGTCAAGTGTAGAA-3′) or negative control probe with rotation for 180 min at 37 °C, followed by addition of streptavidin beads with rotation for 30 min at room temperature. Finally, the protein bound to the beads was dissociated using elution buffer and analyzed by immunoblot assay.

### Deletion of mgU2-30 in hBMECs via CRISPR/Cas9 technology

A three-in-one pYSY-spCas9-sgRNA-Puro vector was obtained from YSY Biotech (Nanjing, China). Human mgU2-30-sgRNA1 (5′- CACCGACTGATCTTTGTAACTATGA-3′) and sgRNA2 (5′- AAACAATCATTTCTGGGCAATGATC-3′) were synthesized and cloned into the vector to generate the pYSY-spCas9-mgU2-30-sgRNA-Puro construct. hBMECs were seeded into six-well plates at a density of 2 × 10^5^ cells per well for 24 h, followed by transient transfection with 2 μg of pYSY-spCas9-mgU2-30-sgRNA-Puro plasmid using the jetPRIME transfection reagent. The cells were incubated at 37 °C with 5% CO_2_ for 24 h, and a fresh medium containing 200 ng/mL puromycin was added and incubated for another 48 h. Cells were then collected, and each single-cell clone was transferred into 96-well plates following a limiting dilution method. Genomic DNA was extracted when the cells were confluent using QuickExtract DNA Extraction Solution (YSY Biotech). Cells with mgU2-30 sequence deletion were validated by PCR using GoldenStar T6 Super PCR Mix (Tsingke Biological Technology, Beijing, China), as shown in Fig. 4d. The primers used for identification were as follows: 5′-TTATGCTGTGGAGGAAGA-3′ (forward) and 5′-ACTGGGAGCCTTTTAAGT-3′ (reverse) (amplicon PCR1), 5′-TGTGGAGGAAGAACATGC-3′ (forward) and 5′-TTCATCATTGCCCAGAAA-3′ (reverse) (amplicon PCR2), 5′-GGGCAATGATGAAAAGGT-3′ (forward) and 5′AGGCTCCCAGTGGAAACA-3′ (amplicon PCR3), and 5′-AGCTGTTTGGCTTCGTAT-3′ (forward) and 5′-CCATCAATTAGGCTTTCA-3′ (reverse) (amplicon PCR4).

### Statistical analysis

Data are expressed as mean ± SD from three independent experiments, and the significance of differences between groups was evaluated using the t-test. * p < 0.05 was considered significant, and ** p < 0.01 and *** p < 0.001 were considered extremely significant. Graphs were plotted and analyzed using GraphPad Prism (version 6.0; GraphPad Software, La Jolla, CA, USA).

## Supplementary Information

**Additional file 1: Table S1.** Primers used for qPCR. **Fig. S1.** Colocalization analysis of mgU2-30 and IRAK1. Quantitative analysis of mgU2-30 and IRAK1 colocalization by Pearson's correlation and Manders' (M1 and M2) colocalization coefficients. Data were presented as mean ± SD from five independent analyses.

## Data Availability

Data supporting the conclusions of this article are presented in this manuscript or Additional file 1.

## Abbreviations

BBB: Blood–brain barrier
BMEC: Brain microvascular endothelial cell
CNS: Central nervous system
DIG: Digoxigenin
FISH: Fluorescence in situ hybridization
GAPDH: Glyceraldehyde 3-phosphate dehydrogenase
hBMEC: Human brain microvascular endothelial cell
IL: Interleukin
IRAK: Interleukin-1 receptor-associated kinase
LncRNA: Long non-coding RNA
MAPK: Mitogen-activated protein kinase
NF-κB: Nuclear factor kappa B
PBS: Phosphate-buffered saline
PAGE: Polyacrylamide gel electrophoresis
qPCR: Quantitative real-time polymerase chain reaction
RAP: RNA antisense purification
RIP: RNA immunoprecipitation
SDS: Sodium dodecyl sulfate
SSC: Saline sodium citrate
TNF: Tumor necrosis factor
