# Supplementary material for: Long non-coding RNA lncC11orf54-1 modulates neuroinflammatory responses by activating NF-κB signaling during meningitic Escherichia coli infection
Source: Mol Brain. 2022 Jan 3;15:4. doi: 10.1186/s13041-021-00890-8 (PMC8722204; doi:10.1186/s13041-021-00890-8)
Supplement: Supplementary file 1 — Additional file 1: Table S1. Primers used for qPCR. Fig. S1. Colocalization analysis of mgU2-30 and IRAK1. Quantitative analysis of mgU2-30 and IRAK1 colocalization by Pearson's correlation and Manders' (M1 and M2) colocalization coefficients. Data were presented as mean ± SD from five independent analyses. [file 13041_2021_890_MOESM1_ESM.docx]

**Table S1. Primers used for qPCR**

| Primer name | Nucleotide sequence (5’-3’) |
| --- | --- |
| lncC11orf54-1-F | GGTGCGTATGTATGTCTATG |
| lncC11orf54-1-R | GCTCAGGTCAAGTGTAGA |
| mgU2-19-F | AATGATGAAACTAGCCAA |
| mgU2-19-R | CATCAGGTAGACTGGAAA |
| mgU2-30-F | GGGCAATGATGAAAAGGT |
| mgU2-30-R | GCTCAGGTCAAGTGTAGAAAC |
| IL-6-F | ACTCACCTCTTCAGAACGAATTG |
| IL-6-R | CCATCTTTGGAAGGTTCAGGTTG |
| TNF-α-F | CGAGTGACAAGCCTGTAG |
| TNF-α-R | GGACCTGGGAGTAGATGA |
| IL-1β-F | ATGATGGCTTATTACAGTGGCAA |
| IL-1β-R | GTCGGAGATTCGTAGCTGGA |
| GAPDH-F | TGCCTCCTGCACCACCAACT |
| GAPDH-R | CGCCTGCTTCACCACCTTC |

**Figure.**


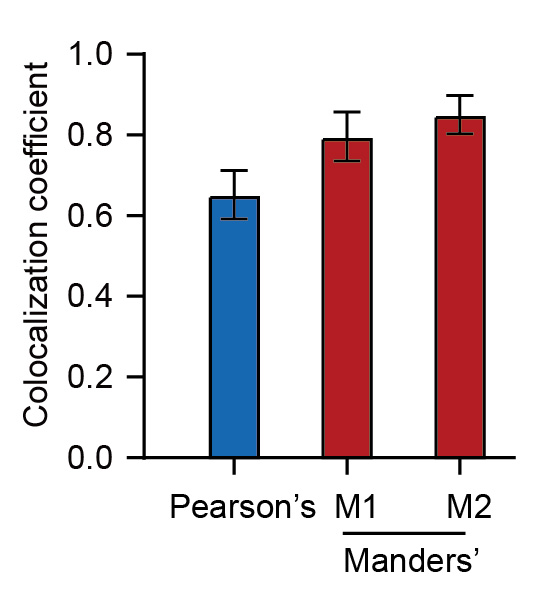


**Figure S1.** Quantitative analysis of mgU2-30 and IRAK1 colocalization by Pearson's correlation and Manders' (M1 and M2) colocalization coefficients. Data were presented as mean ± SD from five independent analyses.
